# Evaluation of Implant Abutment–Soft Tissue Attachment Using 3D Tissue‐Engineered Oral Mucosa: A Systematic Review

**DOI:** 10.1155/ijod/5005401

**Published:** 2026-06-27

**Authors:** Rawan Aboud, Nour Jalaleddine, Dalia Alsadig Mohamed, Moosa Abuzayeda, Keyvan Moharamzadeh

**Affiliations:** ^1^ Department of Restorative Dentistry, Hamdan Bin Mohammed College of Dental Medicine (HBMCDM), Mohammed Bin Rashid University of Medicine and Health Sciences (MBRU), Dubai Health, Dubai, UAE, mbruniversity.ac.ae; ^2^ Department of Restorative Dentistry, School of Clinical Dentistry, University of Sheffield, Sheffield, UK, sheffield.ac.uk

**Keywords:** 3D oral mucosa, abutments, dental implants, soft tissue attachment

## Abstract

**Objective:**

Three‐dimensional (3D) tissue–engineered oral mucosa models offer a physiologically relevant platform to study interactions between implant abutment materials and peri‐implant soft tissues. While osseointegration is well‐characterized, soft tissue sealing, essential for preventing infection and maintaining implant stability, remains thoroughly underexplored. Despite many in vitro studies using these models, a comprehensive evaluation in this field is lacking. This study systematically reviews current evidence to guide future research and improve clinical outcomes.

**Methods:**

PubMed, Scopus, and Embase were searched (31 March 2026) following preferred reporting items for systematic reviews and meta‐analyses (PRISMA) 2020 guidelines, identifying 1551 records. After removing 623 duplicates, 227 records were screened (204 excluded); 23 full texts were assessed (nine excluded), yielding 14 studies using human 3D oral mucosa models (3D‐OMMs) to assess implant abutment interfaces.

**Results:**

All 14 studies used epithelial‐fibroblast cocultures replicating native mucosal architecture. Histological analyses confirmed stratified squamous epithelium formation, with some studies showing epithelial surface maturation via immunohistochemistry. Epithelial interface–associated biological response to different implant surfaces has been assessed using electron microscopy. Surface modifications like anodization, UV treatment, and increased hydrophilicity improved cell adhesion and interface organization. However, permeability and postpull‐out metabolic activity assays suggested that barrier function and residual tissue viability were generally similar across materials, although specific surface treatments improved some outcomes. Molecular studies highlighted that peri‐implant mucosa interactions with oral biofilms are crucial for maintaining or disrupting tissue homeostasis by modulating immune responses.

**Conclusions:**

3D‐OMMs reliably recapitulate peri‐implant tissue responses. Overall, the available in vitro evidence suggests that surface modification and host–microbe interactions may influence mucosal homeostasis and barrier integrity, although findings remain heterogeneous and should be interpreted cautiously.

## 1. Introduction

Different dental implant materials have been developed and tested to optimize osseointegration and increase clinical success rates. However, in addition to these advancements, enhancing the biological soft tissue seal around implants, serving as a crucial barrier against microbial invasion and peri‐implantitis, is critical for achieving favorable long‐term treatment outcomes and preventing implant failure [1]. Historically, researchers have evaluated soft tissue attachment to various abutment materials using multiple biological models, including in vivo animal studies, conventional monolayer (2D) epithelial cell cultures, and, more recently, three‐dimensional (3D) tissue–engineered oral mucosa models [2].

While animal models have provided valuable insights into the peri‐implant environment, they are limited by ethical concerns, high costs, and the lack of direct human applicability [3]. Similarly, 2D monolayer cultures, although accessible and reproducible, fail to replicate the complex multilayer architecture and signaling environment of the native oral mucosa. The oral mucosa naturally comprises a stratified squamous epithelial layer and a connective tissue layer (lamina propria), both critical for realistic modeling of implant–tissue interactions [4]. To overcome these limitations, 3D tissue–engineered oral mucosa models have been developed. These models coculture human keratinocytes and fibroblasts in a multilayer format, successfully mimicking the structural and functional characteristics of native tissue [5, 6]. Emerging approaches, including tissue stem cell treatments, offer potential to further increase soft tissue volume and quality around implants while controlling inflammation levels, as demonstrated in recent systematic reviews on dental implant osseointegration [7]. Additionally, 3D models enable integrating multiple analytical techniques, including histology, immunohistochemistry, electron microscopy, molecular biology, and microbial assays, making them highly versatile for investigating both tissue integration and host–microbe dynamics [8–10]. Given the growing number of studies utilizing these advanced in vitro systems, this systematic review aims to consolidate and evaluate the evidence on implant‐soft tissue interactions using 3D oral mucosa models (3D‐OMMs). By assessing epithelial attachment, tissue response, and microbial influence across different abutment materials and surface modifications, the review seeks to guide future research and improve translational applications in implant dentistry. Recent umbrella reviews highlight complementary clinical strategies. Acerra et al. [11] demonstrated PRF/PRP accelerates soft tissue healing (SMD = 1.24 and *p*  < 0.001), while D’Ambrosio et al. [12] optimized palatal graft harvesting for keratinized mucosa augmentation. Our recent study [13] demonstrated that UV‐photofunctionalized titanium (Ti) surfaces enhance epithelial interface–associated biological response in 3D‐OMMs by 42% compared to machined Ti (*p*  < 0.01), providing direct evidence supporting surface modification strategies evaluated in this systematic review [14].

## 2. Materials and Methods

### 2.1. Protocol and Search Strategy

This systematic review followed the preferred reporting items for systematic reviews and meta‐analyses (PRISMA) guidelines [15].

**Figure 1:**
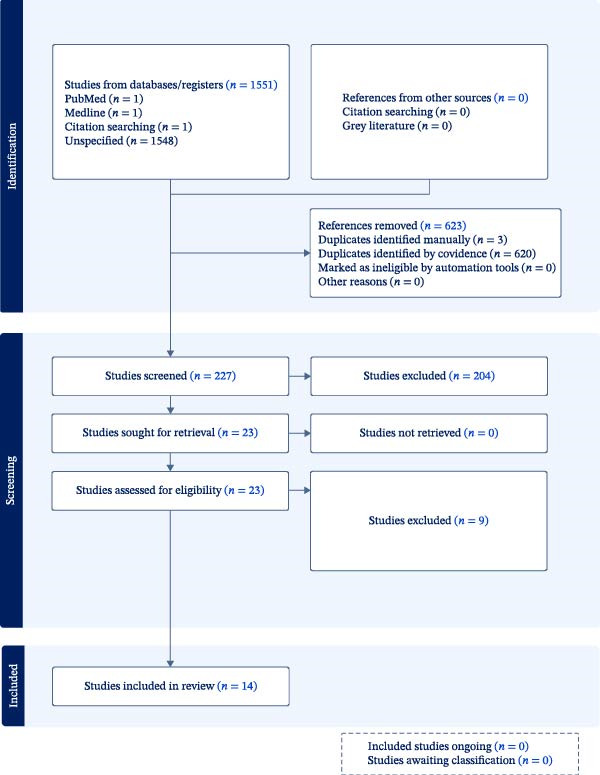
PRISMA flow chart.

Literature searches were conducted across PubMed/MEDLINE, Scopus, Embase, and Google Scholar using the following keywords: 3D oral mucosa, organotypic models, dental implants, abutments, tissue‐engineered models, soft tissue interface, and abutment soft tissue interface. The publication histories of authors for relevant literature and nonscientific sources (Google) were also searched to identify any missed studies. The search for data included studies published from 2010 until March 31, 2026, with full Boolean operators: (“3D Human oral mucosa” OR “organotypic”) AND (“dental implant” OR abutment). The search strategy has been summarized in Supporting Information 1: Table S1. Duplicates of references were removed using EndNote software version 20 (Clarivate, Chandler, AZ). The data collection process was summarized and updated in a flowchart in accordance with PRISMA guidelines (Figure 1) using the Covidence workflow platform (Melbourne, Australia), including manual literature search and selection criteria. The search was restricted to studies published in English. Two authors (R.A. and N.J.) independently reviewed the titles and abstracts to assess their eligibility (κ = 0.87) based on the inclusion criteria. Any disagreements (*n* = 3) were resolved by consulting a third author (K.M.). The full texts of those potentially eligible title abstracts were obtained, and the full texts were independently reviewed by the same authors (R.A. and N.J.). Any disagreement was solved by a discussion, and a third author (K.M.) was consulted in case of doubt. The reference lists of the included reviews were also screened for relevant titles, and the subsequent study screening was performed as already described.

### 2.2. Selection Criteria

#### 2.2.1. Inclusion Criteria

The population, intervention, comparison, outcomes, and studies (PICOS) approach established the inclusion criteria: P (participants): 3D cultured cells or cell lines from donor tissue of human oral mucosa origin; I (intervention): implant and abutment insertion; C (comparison): tissue response/attachment to different types of dental implant and abutment surfaces; O (outcomes): tissue viability, histology appearance, ultrastructural analysis, interface permeability, attachment contour morphology, and inflammatory response; and S (studies): in vitro studies (Table 1).

**Table 1:** Inclusion and exclusion criteria of article selection.

Criteria	Inclusion criteria	Exclusion criteria
Cells at the implant interface	3D cultured cells or cell lines from donor tissue of human oral mucosa origin; human oral mucosa cells (keratinocytes/fibroblasts) form functional tissue‐dental implant contact	Animal cells ONLY (no human cells at the interface)
Study type	Implant and abutment insertion in 3D tissue–engineered model‐in vitro 3D organotypic models	In vivo and clinical studies
Feeder layers	Xenogeneic feeders OK (3T3‐J2 mitomycin C inactivated)	N/a
Language	Publications written in English	Publications not written in English

#### 2.2.2. Exclusion Criteria

The exclusion criteria employed for this study were clinical studies or in vivo studies, as well as studies using animal‐derived primary cells or animal tissue as the functional interface. Xenogeneic feeder layers, such as mitomycin C–inactivated 3T3‐J2 cells, were permitted when used only as nonproliferative support components in standard organotypic protocols, provided that human oral mucosa cells formed the tissue–implant interface and generated the study outcomes [2]. The functional interface was defined as the human oral mucosa cell layer directly contacting the implant surface and contributing to the reported biological outcomes, whereas support components referred to feeder cells or scaffold‐support elements that facilitated tissue formation but were not themselves the measured interface. Furthermore, publications not written in English were also excluded (Table 1).

### 2.3. Data Extraction and Synthesis

A standardized data extraction form was used by two independent reviewers (R.A. and N.J.), and any discrepancies were resolved by discussion, with input from a third author (K.M.) if needed. A narrative synthesis of the data concerning the investigated population, exposure, and outcomes was conducted. Data from the included studies were qualitatively synthesized through descriptive analysis.

### 2.4. Quality Assessment

Two reviewers (R.A. and N.J.) independently assessed the risk of bias for all 14 studies using the QUIN‐12 (risk of bias tool for assessing in vitro studies conducted in dentistry) [16]. The assessment covered 12 domains: clearly stated aims and objectives, sample size calculation, sampling technique, comparison group, methodology, operator details, randomization, outcome measurement, outcome assessor details, blinding, statistical analysis, and presentation of results. Each domain was rated as low risk (1), unclear (0), or high risk (10) based on the clarity and completeness of reporting. Studies were then classified overall as having low, moderate, or high risk of bias according to the number and nature of domains with unclear or missing information. Overall risk of bias was then categorized as low, moderate, or high based on the number and importance of domains.

## 3. Results

The literature search identified 1551 records, of which 623 duplicates were removed. Following title and abstract screening, 227 records were assessed, and 23 full texts were reviewed for eligibility, from which nine full texts were excluded (Supporting Information 2: Table S2). Ultimately, 14 studies met the inclusion criteria and used human 3D‐OMMs to evaluate implant–abutment interfaces. All included studies were in vitro employed epithelial–fibroblast cocultures designed to mimic native oral mucosal architecture (Table 2).

**Table 2:** Summary table of included studies.

Title of study	Author name and year	Biological system	Implant	Biological tests	Evaluation
Development of a novel model for the investigation of the implant‐soft tissue interface	Chai et al., 2010 [4]	3D‐OMM was constructed using HGK and HGF cultured on a skin‐derived scaffold at an air‐liquid interface	1. Polished Ti 2. Machined Ti 3. Sandblasted Ti 4. TiUnite‐treated Ti	1. Histology and histomorphometry analysis 2. Immunohistochemistry 3. Scanning electron microscopy (SEM)	The 3D‐OMM replicated peri‐implant features observed in vivo and may serve as a relevant alternative model for evaluating implant–soft tissue interactions
The biological seal of the implant‐soft tissue interface was evaluated in a tissue‐engineered oral mucosal model	Chai et al., 2012 [17]	3D‐OMM was constructed using TR146 and HGF cultured onto the basement membrane side of the acellular scaffold	1. Polished Ti 2. Machined Ti 3. Sandblasted Ti 4. TiUnite‐treated Ti	1. Histology and histomorphometry analyses 2. Scanning electron microscopy (SEM) 3. Postpull‐out metabolic activity assays 4. Permeability test	The four titanium surface topographies did not produce clear differences in soft tissue attachment. However, although surface roughness may not significantly influence soft tissue attachment, it may still affect plaque accumulation. In general, smooth, polished implant surfaces provide better plaque control
Ultrastructural analysis of implant‐soft tissue interface on a three‐dimensional tissue‐engineered oral mucosal model	Chai et al., 2012 [8]	3D‐OMM was constructed using HGK and HGF cultured on an acellular dermis scaffold	1. Polished Ti 2. Machined Ti 3. Sandblasted Ti 4. TiUnite‐treated Ti	1. Scanning electron microscopy (SEM) 2. Transmission electron microscopy (TEM)	The oral mucosal equivalent formed pocket‐like epithelial attachments on polished, machined, sandblasted, and TiUnite surfaces. Ultrastructural analysis suggested that titanium surface topography had little effect on soft tissue attachment. Electropolishing preserved intact implant–mucosa interfaces for transmission electron microscopy, whereas focused ion beam preparation required further optimization
Contour analysis of an implant‐soft tissue interface	Chai et al., 2013 [18]	3D‐OMM was constructed using TR146 and HGF onto an acellular dermis scaffold	1. Polished Ti 2. Machined Ti 3. Sandblasted Ti 4. Anodized Ti	1. Histology and histomorphometry analysis 2. Immunohistochemistry	The in vitro cell line–based 3D‐OMM formed a peri‐implant‐like epithelium at the implant–soft tissue interface, with no significant differences in interface contour among the four titanium surfaces
Evaluation of a novel oral mucosa in vitro implantation model for analysis of molecular interactions with dental abutment surfaces	Roffel et al., 2019 [19]	3D‐OMM was constructed using KC‐TERT and Fib‐TERT, cultured on a fibroblast‐populated collagen hydrogel	1. Anodized Ti 2. Machined Ti	1. Histology and histomorphometry analyses 2. Immunohistochemistry 3. Scanning electron microscopy (SEM)	The RHG model was the first in vitro 3D model to assess both human epithelial attachment to dental abutments and the expression of protein markers involved in soft tissue attachment and integration. No noticeable difference in epithelial attachment was observed between the two abutments
Commensal and pathogenic biofilms differently modulate peri‐implant oral mucosa in an organotypic model	Ingendoh‐Tsakmakidis et al., 2019 [9]	3D‐OMM was constructed using HGFs on a collagen scaffold with OKF6/TERT‐2 cocultured with and without *S. oris* or *A. actinomycetemcomitans* biofilms	HGF‐colonized Ti disk	1. Histology and histomorphometry analyses 2. Molecular biology	*S. oralis* may support peri‐implant health by promoting a balanced protective response, whereas *A. actinomycetemcomitans* may suppress host defense, potentially facilitating bacterial colonization and subsequent tissue invasion
Implant soft‐tissue attachment using 3D oral mucosal models: a pilot study	Barker et al., 2020 [20]	3D‐OMM was constructed using HGF, OKF6/TERT‐2, and THP‐1 Monocytes and rat‐tail type I collagen	1. SLA and M‐TiZr 2. ZrO2 3. PEEK 4. Machined PEEK	1. Histology and histomorphometry analyses 2. Scanning electron microscopy (SEM) 3. Postpull‐out metabolic activity assays	The inflamed 3D oral mucosal model showed potential as a suitable in vitro system for visualizing and quantifying implant–soft tissue attachment. Greater soft tissue interface–associated biological response was observed on TiZr‐SLA than on TiZr‐M, ceramic, and PEEK surfaces
Biofilm Interactions of *Candida albicans* and Mitis Group Streptococci in a titanium‐mucosal interface model	Souza et al., 2020 [21]	3D‐OMM was constructed of SCC15 collagen scaffold and 3T3 cocultured with monospecies (*C. albicans*) or mixed‐species biofilms (*C. albicans* with different *Streptococcus* species)	Machined Ti discs	1. Histology and histomorphometry analyses 2. Molecular biology 3. Microbiology	Mixed *Candida albicans*–streptococcal biofilms on titanium may contribute to peri‐implant mucosal damage, while mucosal release of Candida‐suppressing factors may help limit fungal overgrowth
Early host–microbe interaction in a peri‐implant oral mucosa‐biofilm model	Mikolai et al., 2020 [10]	3D‐OMM was constructed using HGFs and OKF6/TERT‐2 on a collagen scaffold cocultured with and without multispecies biofilms: *S. oralis*, *A. naeslundii*, *V. dispar*, and *P. gingivalis* biofilm	HGF‐colonized Ti disk	1. Molecular biology 2. Microbiology	The 3D peri‐implant mucosa model showed that early interaction with commensal multispecies biofilm may initially resemble host–microbe homeostasis, but this balance was disrupted after prolonged exposure, leading to tissue damage and enhanced inflammation. These findings improve the understanding of peri‐implant host–microbe interactions and may help guide prevention and treatment strategies for peri‐implant diseases
An in‐vitro analysis of peri‐implant mucosal seal following photofunctionalization of zirconia abutment materials	Razali et al., 2021 [1]	3D‐OMM was constructed using HGK and HGF cultured on an acellular dermal membrane	1. YSZ 2. ATZ 3. CPTi	1. Histology and histomorphometry analyses 2. Atomic force microscopy (AFM) 3. Permeability test	Photo functionalization enhanced the peri‐implant soft tissue biological seal around implant abutment materials, with YSZ showing the most favorable sealing performance among the tested materials
3D engineered human gingiva fabricated with electrospun collagen scaffolds provides a platform for in vitro analysis of gingival seal to abutment materials	Sakulpaptong et al., 2022 [22]	3D‐OMM was constructed using HGF and HGK cultured on four matrices: electrospun collagen, decellularized dermis, type I collagen gels, and released type I collagen gels	1. SLA Ti 2. Machined Ti 3. TiN‐coated Ti 4. PEEK 5. Ceramic	1. Histology and histomorphometry analyses 2. Immunohistochemistry	Electrospun collagen provided a scalable, reproducible, and cost‐effective scaffold for engineering human gingiva with high fidelity to native tissue, and the model was suitable for studying biomaterial–soft tissue interactions at the implant–gingival interface
Contour analysis of three‐dimensional peri‐implant mucosal model as an endpoint analysis of photofunctionalization effects on implant abutment materials	Razali et al., 2023 [23]	3D‐OMM was constructed using HGF and TR146 cultured on an acellular dermal membrane	1. YSZ 2. ATZ 3. CPTi All were tested with and without UV treatment	1. Histology and histomorphometry analyses 2. Scanning electron microscopy (SEM)	UV‐mediated photofunctionalization improved peri‐implant soft tissue morphology by increasing the formation of a nonpocket‐type contour in the 3D peri‐implant mucosal model. YSZ produced a more favorable soft tissue contour than ATZ and titanium
Nanostructured implant‐tissue interface assessment using a three‐dimensional gingival tissue equivalent	Llopis‐Grimalt et al., 2024 [24]	3D‐OMM was constructed using GTE of iHGF and iHGK	1. NN Ti 2. Machined Ti	1. Immunohistochemistry 2. Scanning electron microscopy (SEM) 3. Postpull‐out metabolic activity assays 4. Molecular biology	Nanostructured titanium was biocompatible and promoted collagen alignment closer to the natural soft tissue interface, which may support better sealing
Differential attachment of engineered oral soft tissues to implant surfaces	Jalaleddine et al., 2026 [14]	Three 3D oral mucosal model types were established: epithelium‐only, connective tissue‐only, and full‐thickness models. Inflammation was induced in all models using *E. coli*, LPS, and TNF‐α	1. TiZr‐SLA 2. Machined Ti 3. Machined Zr 4. Polished Zr 5. Machined PEEK rods 6. HA‐Ti 7. HA‐ZrO_2_	1. Histology and histomorphometry analyses 2. Scanning electron microscopy (SEM) 3. Molecular biology 4. Postpull‐out metabolic activity assays	Implant material, surface, and design all affect soft tissue response, with rough TiZr‐SLA favoring connective tissue interface–associated biological response and smooth commercial abutments showing the best overall integration

**Figure 2:**
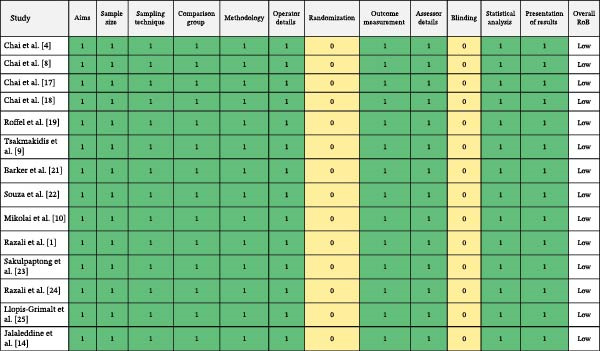
Risk of bias assessment of the included studies using the QUIN tool. Green indicates low risk (1), yellow indicates unclear risk (0), and red indicates high risk (10).

Risk of bias assessment was performed using the QUIN tool. Overall, most included studies were judged to have a low‐to‐moderate risk of bias, as most domains were adequately reported across the papers. The studies generally provided clear aims, appropriate comparison groups, detailed methodologies, outcome measurement methods, statistical analyses, and well‐presented results. The main domains that were less consistently reported were randomization and blinding, which were unclear in several studies. However, because these items are not always explicitly described in vitro 3D oral mucosal research, the remaining methodological domains were well reported. Thus, the overall risk of bias for the included studies was considered low to moderate, primarily because randomization and blinding were incompletely reported in several studies (Figure 2).

The following sections provide in‐depth and point‐by‐point analyses of each biological endpoint assessed in these studies.

### 3.1. Histological and Histomorphometric Analyses

Histological and histomorphometric analyses are widely used to assess the structural integrity, epithelial stratification, and connective tissue organization of engineered oral mucosa. Between 2010 and 2026, significant advancements have been made in 3D oral mucosal modeling. As shown in Table 2, early studies by Chai et al. [4, 17, 18] consistently demonstrated the formation of well‐organized stratified squamous epithelium, typically 4–6 cell layers thick and measuring 50–100 μm, with both pocket and nonpocket epithelial attachments observed on Ti surfaces. Despite these findings, no clear correlation between epithelial attachment and implant surface topography was established [4, 17, 18]. Roffel et al. [19] identified sulcus‐like epithelial narrowing near abutments, while Barker et al. [20] introduced an inflammatory component, incorporating fibroblasts and monocytes. Later studies by Razali et al. [1, 23] and Sakulpaptong et al. [22] expanded material evaluation to zirconia, polyether ether ketone (PEEK), and ceramics, documenting key developmental milestones such as epithelial and connective layer formation by day 10 and stratum corneum emergence by day 7 (Table 3). Engineered gingiva consistently lacked rete ridges and had thinner epithelium than native tissue. Histological assessments also explored microbial interactions: Ingendoh‐Tsakmakidis et al. [9] found minimal histological changes after biofilm exposure, whereas Souza et al. [21] observed direct fungal and mixed biofilm formation on mucosal surfaces. They highlighted fungal cell detachment from the Ti surface and the formation of biofilms directly on the mucosal tissue during coincubation (Table 3) [9, 21]. The included study by Jalaleddine et al. [14] has recently demonstrated a stratified multilayered oral epithelium with underlying connective tissue containing viable fibroblasts and THP‐1 monocytes in a 3D oral mucosal model, exhibiting histological features closely resembling native gingival mucosa architecture. Collectively, these findings demonstrate considerable progress in 3D oral mucosa modeling, enabling detailed characterization of epithelial and connective tissue architecture across diverse implant materials. Despite replicating key features of native mucosa, engineered models exhibit limitations such as the absence of rete ridges and thinner epithelium. Importantly, integration of immune components and microbial challenges has begun to elucidate complex host–material–biofilm interactions critical for peri‐implant tissue homeostasis. Continued refinement of these models is essential to better mimic in vivo conditions and advance understanding of soft tissue integration and pathogenesis around implant surfaces.

**Table 3:** Histological and histomorphometric analyses highlight tissue response, epithelial stratification, and connective tissue remodeling around different abutment materials.

Biological test: histology and histomorphometry analyses				
Article	Biological system	Implant	Methods	Results
Chai et al. [4]	3D‐OMM was constructed using HGK and HGF cultured on a skin‐derived scaffold at an air‐liquid interface	1. Polished Ti 2. Machined Ti 3. Sandblasted Ti 4. TiUnite‐treated Ti	Light microscope—H&E	The study revealed a well‐developed stratified squamous epithelium with 4–6 cell layers. Histological sections showed varying epithelial stratification thickness, confirming the successful development of the organotypic culture model. Two types of epithelial attachments (pocket and nonpocket) were identified on all Ti surfaces. Still, no clear correlation was found between attachment pattern and surface topography, suggesting surface characteristics may not directly influence attachment type
Chai et al. [17]	3D‐OMM was constructed using TR146 and HGF cultured onto the basement membrane side of the acellular scaffold	1. Polished Ti 2. Machined Ti 3. Sandblasted Ti 4. TiUnite‐treated Ti	Light microscope—H&E	The oral mucosa model (OMM) histological analysis revealed a stratified squamous epithelium 50–100 μm thick, comprising 4–6 cell layers. The OMM generally maintained its structure but showed localized damage at the interface and clamped areas, especially after titanium disc removal. These spots exhibited disrupted epithelium, unlike the rest of the well‐formed tissue
Chai et al. [18]	3D‐OMM was constructed using TR146 and HGF onto an acellular dermis scaffold	1. Polished Ti 2. Machined Ti 3. Sandblasted Ti 4. Anodized Ti	Light microscope—H&E	The OMM replicated the structure of normal oral mucosa, featuring a stratified squamous epithelium that closely mimicked natural tissue characteristics
Roffel et al. [19]	3D‐OMM was constructed using KC‐TERT and Fib‐TERT, cultured on a fibroblast‐populated collagen hydrogel	1. Anodized Ti 2. Machined Ti	Light microscope—H&E	A sulcus‐like area was observed near the upper RHG surface, with epithelium growing downwards along abutments, tapering from 7–9 to 1–2 cell layers. After 10 days, sulcus depths were 143 ± 42 μm for the anodized surface and 148 ± 55 μm for the machined surface, notably shallower than typical human clinical studies
Ingendoh‐Tsakmakidis et al. [9]	3D‐OMM was constructed using HGFs on a collagen scaffold with OKF6/TERT‐2 cocultured with and without *S. oris* or *A. actinomycetemcomitans* biofilms	HGF‐colonized Ti disk	Light microscope—H&E	Peri‐implant mucosa exposed to *S. oralis* or *A. actinomycetemcomitans* biofilm for 24 h showed an intact implant‐mucosa interface. The *S. oralis* biofilm caused slight loosening of the epithelium directly at the implant, while the epithelium farther from the implant remained histologically similar to the control tissue. The effect of *S. oralis* biofilm was limited to the implant‐mucosa interface. *A. actinomycetemcomitans* biofilm exposure had no visible histological impact on the mucosa
Barker et al. [20]	3D‐OMM was constructed using HGF, OKF6/TERT‐2, and THP‐1 Monocytes and rat‐tail type I collagen	1. SLA and M TiZr 2. ZrO_2_ 3. PEEK 4. Machined PEEK	Light microscope—H&E	A multilayered, stratified oral epithelium and an underlying connective tissue are present, with fibroblasts and monocytes
Souza et al. [21]	3D‐OMM was constructed using SCC15 collagen scaffold and 3T3 cocultured with monospecies (*C. albicans*) or mixed‐species biofilms (*C. albicans* with different *Streptococcus* species)	Machined Ti discs	Fluorescence staining techniques	Fungal cells dispersed from the titanium and formed biofilms directly on the mucosal surface during coincubation
Razali et al. [1]	3D‐OMM was constructed using HGK and HGF cultured on an acellular dermal membrane	1. YSZ 2. ATZ 3. CPTi	Light microscope—H&E	Well‐developed stratified squamous epithelium comprising four to six epithelial cell layers. The histological sections of punched tissues grown alongside the 3D‐PIMM displayed epithelial stratification of varying thicknesses. The presence of epithelial layers on the acellular membrane confirmed the successful development of the organotypic culture model
Sakulpaptong et al. [22]	3D‐OMM was constructed using HGF and HGK cultured on four matrices: electrospun collagen, decellularized dermis, type I collagen gels, and released type I collagen gels	1. SLA Ti 2. Machined Ti 3. TiN‐coated Ti 4. PEEK 5. Ceramic	Light microscope—H&E	Engineered gingiva developed two distinct layers—epithelium and connective tissue—by day 10. Densely packed basal keratinocytes formed at the epithelium‐stroma junction by day 10–14. The stratum corneum appeared by day 7 and thickened over time. Compared to native gingiva, the engineered gingiva lacked rete ridges and had an approximately one‐third‐thick epithelium
Razali et al. [23]	3D‐OMM was constructed using HGF and TR146 cultured on an acellular dermal membrane	1. YSZ 2. ATZ 3. CPTi All were tested with and without UV treatment	Light microscope—H&E	Epithelial cell migration and attachment to the implant interface across all materials. This attachment and proliferation led to the formation of pocket and nonpocket tissue contours
Jalaleddine et al. [14]	3D‐OMM was composed of stratified oral keratinocytes (OKF6/TERT‐2) cultured atop a collagen‐based connective tissue populated with HGFs and THP‐1 monocytes	1. TiZr‐SLA 2. TiZr‐M 3. ZrO_2_‐M 4. PEEK‐M	Light microscope—H&E	Histology revealed well‐stratified oral epithelium with underlying connective tissue containing viable fibroblasts and THP‐1 monocytes, exhibiting architecture closely mimicking native gingival mucosa

*Note:* 3T3, mouse embryonic fibroblast cell line; Fib‐TERT, fibroblast cell line with telomerase reverse transcriptase; KC‐TERT, keratinocyte cell line with telomerase reverse transcriptase; OKF6/TERT‐2, oral keratinocyte cell line with telomerase reverse transcriptase; TR146, human buccal squamous cell carcinoma cell line.

Abbreviations: 3D‐OMM, three‐dimensional oral mucosa model; 3D‐PIMM, three‐dimensional peri‐implant mucosa model; *A. actinomycetemcomitans*, *Aggregatibacter actinomycetemcomitans*; ATZ, alumina‐toughened zirconia; CPTi, commercially pure titanium; H&E, hematoxylin and eosin; HGFs, human gingival fibroblasts; HGKs, human gingival keratinocytes; M, machined surface; OMM, oral mucosa model; PEEK, polyether ether ketone; RHG, reconstructed human gingiva; *S. oralis*, *Streptococcus oralis*; SCC15, squamous cell carcinoma cell line 15; SLA, sandblasted, large‐grit, acid‐etched surface; Ti, titanium; TiN, titanium nitride–coated titanium; TiZr, titanium‐zirconium alloy; YSZ, yttria‐stabilized zirconia; ZrO_2_, zirconium dioxide.

### 3.2. Immunohistochemistry

Immunohistochemical analyses across multiple studies have comprehensively examined epithelial differentiation, basement membrane formation, and extracellular matrix organization in 3D‐OMMs cultured on various implant surfaces. As shown in Table 4, Chai et al. [4] demonstrated that suprabasal epithelial cells on polished, machined, sandblasted, and TiUnite Ti surfaces exhibited strong cytokeratin‐13 (CK13) but weak cytokeratin‐10 (CK10) expression, indicative of a predominantly nonkeratinized epithelial phenotype. Conversely, Chai et al. [18], using an oral mucosa model (OMM) cell line on similar Ti surfaces, reported strong CK10 and weak CK13 expression, reflecting a keratinized epithelial phenotype more typical of normal oral mucosa (Table 4). Roffel et al. [19] observed altered keratin profiles in reconstructed human gingiva (RHG) adjacent to anodized and machined Ti abutments, noting loss of keratin 4 (K4) and increased keratin 19 (K19) expression characteristic of junctional epithelium–like phenotype at the implant interface; basement membrane proteins collagen IV and laminin‐5 were present at the epithelium–hydrogel interface but absent on outer epithelium near abutments (Table 4). Sakulpaptong et al. [22] reported that engineered gingiva grown on various matrices and implant materials—including SLA Ti, machined Ti, TiN‐coated Ti, PEEK, and ceramics—developed key epithelial markers (cytokeratin‐4, ‐5, and ‐10), basement membrane proteins (laminin‐332 and collagen IV), and fibroblast populations comparable to native gingiva after 10–14 days; however, collagen type I deposition was confined to a thin layer beneath the epithelium, contrasting with the dense basket–weave collagen matrix and vascularization found in native tissue. Most recently, Llopis‐Grimalt et al. [24] demonstrated that implant surface topography influences collagen fiber orientation in 3D‐OMMs derived from iHGF and iHGK cells, with non‐nanostructured Ti (NN Ti) surfaces promoting predominantly perpendicular collagen alignment, in contrast to parallel fiber orientation on machined surfaces, a difference substantiated by quantitative immunostaining (Table 4).

**Table 4:** Immunohistochemistry analysis showcasing protein expression and cellular interactions around different abutment materials.

Biological test: immunohistochemistry				
Article	Biological system	Implant	Methods	Results
Chai et al. [4]	3D‐OMM was constructed using HGK and HGF cultured on a skin‐derived scaffold at an air‐liquid interface	1. Polished Ti 2. Machined Ti 3. Sandblasted Ti 4. TiUnite‐treated Ti	Light microscope—CK10 and CK13	The suprabasal cell layer exhibited a strong CK13 expression but showed weak CK10 expression
Chai et al. [18]	3D‐OMM was constructed using TR146 and HGF onto an acellular dermis scaffold	1. Polished Ti 2. Machined Ti 3. Sandblasted Ti 4. Anodized Ti	Light microscope—H&E, CK10, and CK13	OMM cell line strongly expressed CK10 (keratinized epithelial marker) but weakly expressed CK13 (nonkeratinized epithelial marker) in the suprabasal layer. These findings indicate that the OMM cell line resembles normal keratinized oral epithelium
Roffel et al. [19]	3D‐OMM was constructed using KC‐TERT and Fib‐TERT, cultured on fibroblast‐populated collagen hydrogel	1. Anodized Ti 2. Machined Ti	Light microscope‐ H&E, keratin 4, keratin 19, Ki67, laminin‐5, and collagen IV	The RHG epithelium near the abutments lacked K4 expression and exhibited strong K19 expression a pattern characteristic of the junctional epithelium. In contrast, regenerating epithelium in wounded RHG without implants showed strong K4 expression in all cells and K19 only in basal cells, indicating a nonjunctional epithelium profile. Collagen IV and laminin‐5 also formed a distinct basement membrane between the down‐growing epithelium and hydrogel but were absent on the outer epithelium near the abutments
Sakulpaptong et al. [22]	3D‐OMM was constructed using HGF and HGK cultured on four matrices: electrospun collagen, decellularized dermis, type I collagen gels, and released type I collagen gels	1. SLA Ti 2. Machined Ti 3. TiN‐coated Ti 4. PEEK 5. Ceramic	Polarized light microscopy—H&E, laminin, collagen IV, and Picrosirius red Polarized light microscopy– epifluorescence microscopy	Engineered gingiva developed key structural and protein components like native human gingiva over 10–14 days in culture. Positive staining for cytokeratin‐5, laminin‐332, collagen type IV, cytokeratin‐10, and cytokeratin‐4 was observed in engineered and native gingiva, indicating proper epithelial development and basement membrane formation. Though their distribution differed, fibroblasts (TE‐7 + cells) were present in both types. While native gingiva showed large collagen type I bundles with a basket–weave morphology, the engineered gingiva only developed a small layer of collagen type I fibers at the epithelium‐connective tissue junction after 14 days. Blood vessels were present in native gingiva but absent in the engineered tissue
Llopis‐Grimalt et al. [24]	3D‐OMM was constructed using GTE of iHGF and iHGK	1. NN Ti 2. Machined Ti	Confocal microscope‐immunostaining	Distinct collagen fiber orientations on titanium surfaces. (NN) Surfaces showed predominantly perpendicular collagen fiber alignment, while machined surfaces displayed parallel fiber orientation. Quantitative analysis confirmed a significantly higher proportion of perpendicularly oriented collagen on (NN) surfaces

*Note:* K19, cytokeratin‐19 (junctional epithelium marker); K4, cytokeratin‐4 (differentiation marker); KC‐TERT, keratinocyte cell line with telomerase reverse transcriptase; TR146, human buccal squamous cell carcinoma cell line.

Abbreviations: 3D‐OMM, three‐dimensional oral mucosa model; CK10, cytokeratin‐10 (keratinized epithelial marker); CK13, cytokeratin‐13 (nonkeratinized epithelial marker); Fib‐TERT, fibroblast cell line with telomerase reverse transcriptase; H&E, hematoxylin and eosin staining; HGFs, human gingival fibroblasts; LPS, lipopolysaccharide; NN Ti, nanostructured titanium; OMM, oral mucosa model; PEEK, polyether ether ketone; RHG, reconstructed human gingiva; SLA Ti, sandblasted, large‐grit, acid‐etched titanium; Ti, titanium; TiN, titanium nitride–coated titanium.

Collectively, these immunohistochemical studies reveal that 3D‐OMMs recapitulate critical epithelial and connective tissue characteristics, where differentiation markers and basement membrane proteins reflect tissue‐specific phenotypes modulated by implant surface properties. Implant topography significantly influences epithelial keratin expression patterns and collagen fiber organization, suggesting that targeted surface modifications can guide peri‐implant soft tissue architecture. Nonetheless, current engineered models exhibit limitations, including reduced collagen matrix complexity and absence of vascularization, underscoring the need for further refinement to more closely mimic native peri‐implant mucosa and improve clinical relevance.

### 3.3. Electron Microscopy and Atomic Force Microscopy (AFM)

Electron microscopy techniques, such as scanning electron microscopy (SEM) and transmission electron microscopy (TEM), were used to closely examine how epithelial and connective tissue cells interact with different implant surfaces, providing detailed visualization of cell attachment and interface characteristics (Tables 5 and 6). AFM was also applied to measure surface roughness at the nanoscale, helping to understand how subtle topographical differences might influence biological responses and soft tissue integration (Table 7).

**Table 5:** Scanning electron microscopy (SEM) illustrates surface morphology and cellular interactions with different abutment materials.

Biological test: scanning electron microscopy (SEM)				
Article	Biological system	Implant	Methods	Results
Chai et al. [4]	3D‐OMM was constructed using HGK and HGF cultured on a skin‐derived scaffold at an air‐liquid interface	1. Polished Ti 2. Machined Ti 3. Sandblasted Ti 4. TiUnite‐treated Ti	Conventional SEM imaging	Evidence indicates that cells adhered to the machined titanium surface in the exposed area. This suggests that cells from the 3D‐OMM form attachments on the titanium surface
Chai et al. [8]	3D‐OMM was constructed using HGK and HGF cultured on an acellular dermis scaffold	1. Polished Ti 2. Machined Ti 3. Sandblasted Ti 4. TiUnite‐treated Ti	Conventional SEM imaging	The titanium oxide layer was very thin and not visible in the electron micrograph, unlike TiUnite, which displayed a thick, porous titanium oxide layer ranging from 1 to 6 µm thick. Epithelial cells remained attached to titanium surfaces regardless of roughness
Chai et al. [17]	3D‐OMM was constructed using TR146 and HGF cultured onto the basement membrane side of the acellular scaffold	1. Polished Ti 2. Machined Ti 3. Sandblasted Ti 4. Anodized Ti	Conventional SEM imaging	Epithelial cells attach to all Ti surfaces, regardless of surface roughness. Cells adapted to the underlying topography appear flat and spread on polished and machined surfaces, irregular on sandblasted surfaces, and grow over the “volcano‐like” structures on anodized (TiUnite) surfaces. Additionally, cells were observed not only at the interface but also above it, where they appeared more flattened and formed a monolayer
Roffel et al. [19]	3D‐OMM was constructed using KC‐TERT and Fib‐TERT, cultured on a fibroblast‐populated collagen hydrogel	1. Anodized Ti 2. Machined Ti	Conventional SEM imaging	A continuous layer of epithelial cells covered machined and anodized abutment surfaces At higher magnifications, keratinocytes were seen extending from the migrating epithelial front onto the abutment surfaces
Barker et al. [20]	3D‐OMM was constructed using HGF, OKF6/TERT‐2, and THP‐1 Monocytes and rat‐tail type I collagen	1. SLA TiZr 2. M TiZr 3. ZrO_2_ 4. PEEK 5. Machined PEEK	Conventional SEM imaging	SEM micrographs reveal distinct differences in oral mucosal cell attachments across implant surfaces. The TiZr‐SLA surface stands out with its unique honeycomb structure, while TiZr‐M, ZrO_2_‐M, and PEEK‐M surfaces exhibit similar smooth characteristics with elongated grooves from manufacturing. Cell attachment potential is evident on all implants, but cell morphology varies. Cells appear flat on TiZr‐M, ZrO_2_‐M, and machined PEEK surfaces, whereas they adopt a more three‐dimensional shape on the TiZr‐SLA surface
Razali et al. [23].	3D‐OMM was constructed using HGF and TR146 cultured on an acellular dermal membrane	1. YSZ 2. ATZ 3. CPTi All were tested with and without UV treatment	Conventional SEM imaging	Distinguishing epithelial cells from fibroblasts based on morphology was challenging. Epithelial cells appeared rounded with blebs and microvilli, whereas fibroblasts were spindle‐shaped and elongated. Regardless of treatment, both cell types adhered well to the surfaces, but epithelial cells showed greater attachment on UV‐treated surfaces
Llopis‐Grimalt et al. [24]	3D‐OMM was constructed using GTE of iHGF and iHGK	1. NN Ti 2. Machined Ti	SBF‐SEM	Gingival fibroblasts were showing healthy elongated morphology with a well‐defined cell nucleus and organelles for both implant groups ‐The soft tissue–implant interface has been characterized as connective tissue similar to scar tissue, with collagen fibers predominantly aligned parallel to the implant surface without forming an attachment to it
Jalaleddine et al. [14]	3D‐OMM was composed of stratified oral keratinocytes (OKF6/TERT‐2) cultured atop a collagen‐based connective tissue populated with HGFs and THP‐1 monocytes	1. TiZr‐SLA 2. TiZr‐M 3. ZrO_2_‐M 4. PEEK‐M	Conventional SEM imaging	SEM imaging postpull test revealed abundant cell retention on rough TiZr‐SLA surfaces, contrasting with sparser, patchier coverage on smoother machined TiZr‐M, ZrO_2_‐M, and PEEK‐M surfaces featuring manufacturing grooves. All surfaces supported cell adhesion

*Note:* KC‐TERT, keratinocyte cell line with telomerase reverse transcriptase; OKF6/TERT‐2, oral keratinocyte cell line with telomerase reverse transcriptase; THP‐1, human monocytic cell line; TiUnite, surface modification technique for titanium implants; TR146, human buccal squamous cell carcinoma cell line.

Abbreviations: 3D‐OMM, three‐dimensional oral mucosa model; 3D‐GTE, three‐dimensional gingival tissue equivalent; ATZ, alumina‐toughened zirconia; CPTi, commercially pure titanium; Fib‐TERT, fibroblast cell line with telomerase reverse transcriptase; HGFs, human gingival fibroblasts; HGKs, human gingival keratinocytes; iHGFs, immortalized human gingival fibroblasts; iHGKs, immortalized human gingival keratinocytes; LPS, lipopolysaccharide; M, machined surface; M TiZr, machined titanium–zirconium alloy; NN Ti, nanostructured titanium; OMM, oral mucosa model; PEEK, polyether ether ketone; SBF‐SEM, serial block‐face scanning electron microscopy; SLA TiZr, sandblasted, large‐grit, acid‐etched titanium–zirconium alloy; Ti, titanium; TiN, titanium nitride–coated titanium; TiZr, titanium‐zirconium alloy; TNF‐α, tumor necrosis factor alpha; YSZ, yttria‐stabilized zirconia; ZrO_2_, zirconium dioxide.

**Table 6:** Transmission electron microscopy (TEM) provides detailed insights into the ultrastructural organization and cellular interactions within the 3D oral mucosa model around different abutment materials.

Biological test: transmission electron microscopy (TEM)				
Article	Biological system	Implant	Methods	Results
Chai et al. [8]	3D‐OMM was constructed using HGK and HGF cultured on an acellular dermis scaffold	1. Polished Ti 2. Machined Ti 3. Sandblasted Ti 4. TiUnite‐treated Ti	Focus on beam (Ti remained) and electropolishing (Ti removed) techniques	OMM formed attachments to polished, machined, sandblasted, and TiUnite titanium surfaces via hemidesmosome‐like structures. The epithelial layer consisted of 3–5 interconnected cells with additional features such as membrane‐coating granules and glycogen deposits. The basement membrane exhibited well‐defined anchoring structures, while the connective tissue contained abundant, cross‐linked collagen fibers

*Note:* TiUnite, surface modification technique for titanium implant.

Abbreviations: 3D‐OMM, three‐dimensional oral mucosa model; HGFs, human gingival fibroblasts; HGKs, human gingival keratinocytes; OMM, oral mucosa model; Ti, titanium.

**Table 7:** Atomic Force Microscopy (AFM) analysis of surface topography, roughness, and nanoscale interactions between different abutment materials.

Biological test: atomic force microscopy (AFM)			
Article	Biological system	Implant	Results
Razali et al. [1]	3D‐OMM was constructed using HGK and HGF cultured on an acellular dermal membrane	1. YSZ 2. ATZ 3. CPTi	All material surfaces were classified within the smooth surface range (Sa < 0.5 μm), with no statistically significant differences observed between the various material types (*p* > 0.05)

*Note:* Sa, surface roughness parameter.

Abbreviations: 3D‐OMM, three‐dimensional oral mucosa model; ATZ, alumina‐toughened zirconia; CPTi, commercially pure titanium; HGFs, human gingival fibroblasts; HGKs, human gingival keratinocytes; YSZ, yttria‐stabilized zirconia.

SEM analyses across multiple studies have demonstrated consistent cell attachment and varying morphological adaptations of oral mucosal cells to diverse implant surfaces within 3D‐OMMs. As seen in Table 5, Chai et al. [4, 8, 17] showed that epithelial cells adhered to polished, machined, sandblasted, and TiUnite‐treated Ti surfaces regardless of surface roughness, with cells adapting morphologically flattened and spread on smoother surfaces, irregular on sandblasted surfaces, and conforming to the “volcano‐like” pores characteristic of anodized TiUnite surfaces. Roffel et al. [19] confirmed a continuous epithelial cell layer covering both machined and anodized Ti abutments, highlighting keratinocyte migration and filopodia extension indicative of strong epithelial integration. Barker et al. [20] extended the assessment to different implant materials, observing that rougher TiZr‐SLA surfaces supported 3D cell morphology, whereas smoother TiZr‐M, ZrO_2_, and PEEK surfaces promoted flatter cell shapes. Razali et al. [23] further identified enhanced epithelial cell attachment on UV‐treated surfaces across yttria‐stabilized zirconia (YSZ), alumina‐toughened zirconia (ATZ), and commercially pure Ti (CPTi). Llopis‐Grimalt et al. [24] applied serial block‐face SEM (SBF‐SEM) to reveal healthy gingival fibroblast morphology on NN Ti and machined Ti surfaces; however, the soft tissue–implant interface exhibited scar tissue–like characteristics, with collagen fibers aligned parallel to the implant surface rather than forming direct attachments. The included study by Jalaleddine et al. [14] revealed extensive cell retention on rough TiZr‐SLA surfaces postpull test, contrasting with patchier coverage on machined TiZr‐M, ZrO_2_‐M, and PEEK‐M surfaces (Table 5).

Upon the use of TEM, Chai et al. [8] provided ultrastructural insights, demonstrating that oral mucosal equivalents formed hemidesmosome‐like attachments to polished, machined, sandblasted, and TiUnite Ti surfaces, with a well‐organized basement membrane and abundant cross‐linked collagen fibers in the connective tissue, suggesting robust epithelial–matrix integration (Table 6). While using AFM analysis, Razali et al. [1] were able to characterize the surface topography of various abutment materials, including YSZ, ATZ, and CPTi, as uniformly smooth (Sa<0.5 µm) without significant differences in roughness, indicating comparable nanoscale surface environments for cellular interactions and underscoring that surface chemistry and modification, rather than roughness alone, may play pivotal roles in influencing cell behavior (Table 7). Collectively, these imaging and surface characterization studies reveal that implant material and surface modifications significantly influence peri‐implant soft tissue interactions. While epithelial cells consistently adhere to a range of Ti and ceramic surfaces, surface topography and treatments, such as anodization, sandblasting, and UV irradiation, modulate cell morphology, postpull‐out metabolic activity, and extracellular matrix organization. Despite healthy fibroblast morphology at the implant interface, collagen fibers often align parallel to the surface, resembling scar tissue and indicating a lack of direct connective tissue attachment. The smoothness of implant materials suggests that biochemical surface properties are critical in directing cellular responses. These findings underscore the importance of tailored surface engineering strategies to enhance soft tissue integration and achieve durable peri‐implant seal formation.

### 3.4. Postpull‐Out Metabolic Activity and Permeability Assessment

Although some studies involved specimen removal from the implant surface, the assays reported afterward were metabolic viability assays and not direct mechanical pull‐force measurements. Postpull test metabolic assays and residual cell viability results from multiple studies (Table 8) underscore the impact of implant surface properties on cell interface–associated biological response and viability. Chai et al. [17] observed no significant differences in cell attachment among polished, machined, sandblasted, anodized, and TiUnite‐treated Ti surfaces, with permeability results not correlating with Alamar Blue assay outcomes. In contrast, Barker et al. [20] reported significantly higher cell viability on SLA‐treated TiZr surfaces compared to machined TiZr, ZrO_2_, and PEEK using the PrestoBlue assay, suggesting that rougher surfaces enhance cellular interface–associated biological response. Similarly, Llopis‐Grimalt et al. [24] found that both NN Ti and machined Ti surfaces supported high cell viability with no significant cytotoxicity. Jalaleddine et al. [14] employed a pull test with PrestoBlue viability assessment using a 3D oral mucosal model containing fibroblasts, keratinocytes, and THP‐1 monocytes. Commercial Ti and zirconia healing abutments, along with experimental TiZr‐SLA and ZrO_2_‐P rods, demonstrated superior soft tissue viability postpull test (*p*  < 0.05), while TiZr‐M, ZrO_2_‐M, and PEEK‐M exhibited significantly lower viability in descending order. These results suggest that roughened SLA surfaces and commercial abutments are associated with higher postpull‐out tissue viability and metabolic activity (Table 8).

**Table 8:** Postpull‐out cell viability and metabolic activity assessment of different abutment materials.

Biological test: postpull‐out metabolic activity assays				
Article	Biological system	Implant	Methods	Results
Chai et al. [17]	3D‐OMM was constructed using TR146 and HGF cultured onto the basement membrane side of the acellular scaffold	1. Polished Ti 2. Machined Ti 3. Sandblasted Ti 4. TiUnite‐treated Ti	Viability test‐ Alamar Blue assay	The number of cells remaining attached across the four types of Ti surface topographies was not significantly different
Barker et al. [20]	3D‐OMM was constructed using HGF, OKF6/TERT‐2, and THP‐1 Monocytes and rat‐tail type I collagen	1. SLA and M TiZr 2. ZrO_2_ 3. PEEK 4. Machined PEEK	Viability test‐ PrestoBlue assay	The results demonstrate an almost two‐fold, statistically significant increase in cell viability on the TiZr‐SLA rods compared to the TiZr‐M, ZrO_2_‐M, and PEEK‐M rods (*p* < 0.05)
Llopis‐Grimalt et al. [24]	3D‐OMM was constructed using GTE of iHGF and iHGK	1. NN Ti 2. Machined Ti	Viability test‐ MTT assay	The two different implant surfaces were biocompatible, with all tissues presenting high levels of viability
Jalaleddine et al. [14]	Three types of models: (1) epithelium‐only models composed of only stratified oral keratinocytes (OKF6/TERT‐2) cultured atop a collagen‐based connective tissue (2) Connective tissue‐only models embedded with HGFs and THP‐1 monocytes (3) Full thickness: 3D‐OMM composed of stratified oral keratinocytes (OKF6/TERT‐2) cultured atop a collagen‐based connective tissue populated with HGFs and THP‐1 monocytes	1. TiZr‐SLA 2. TiZr‐M 3. ZrO_2_‐M 4. ZrO‐P 5. PEEK‐M 6. Commercially available: HA‐Ti and HA‐ZrO_2_	Viability‐PrestoBlue assay	Analysis demonstrated significantly enhanced epithelial attachment on TiZr‐SLA, ZrO_2_‐P, and PEEK‐M surfaces versus ZrO_2_‐M and TiZr‐M (*p* < 0.05) TiZr‐SLA exhibited superior connective tissue attachment, particularly outperforming ZrO_2_‐M (*p* = 0.0489) and ZrO_2_‐P (*p* = 0.027) Commercial Ti and ZrO_2_ healing abutments, along with TiZr‐SLA and ZrO_2_‐P experimental rods, demonstrated superior overall soft tissue viability (full thickness) postpull‐out test (*p* < 0.05). TiZr‐M, ZrO_2_‐M, and PEEK‐M exhibited significantly lower viability

*Note:* Gie‐No3B11, gingival epithelial cell line; hTERT, human telomerase reverse transcriptase‐immortalized cell line; MTT, cell viability assay measuring metabolic activity; OKF6/TERT‐2, oral keratinocyte cell line with telomerase reverse transcriptase; THP‐1, human monocytic cell line; TiUnite, surface modification technique for titanium implants; TR146, human buccal squamous cell carcinoma cell line.

Abbreviations: 3D‐OMM, three‐dimensional oral mucosa model; GTE, gingival tissue equivalent; HGFs, human gingival fibroblasts; LPS, lipopolysaccharide; M, machined surface; NN Ti, nanostructured titanium; PEEK, polyether ether ketone; SLA, sandblasted, large‐grit, acid‐etched surface; Ti, titanium; TiZr, titanium‐zirconium alloy; TNF‐α, tumor necrosis factor alpha; ZrO_2_, zirconium dioxide.

Permeability tests (Table 9) further highlight material‐ and treatment‐dependent differences in barrier integrity. Chai et al. [17] demonstrated that although initial permeability rates varied, tracer flux stabilized across all Ti surfaces after 2 h, indicating consistent barrier protection regardless of surface topography. In contrast, Razali et al. [1] showed that UV treatment significantly reduced permeability across all tested materials, including YSZ, ATZ, and CPTi, enhancing barrier properties compared to untreated surfaces. Moreover, treated groups exhibited more effective permeability reductions than nontreated Ti, emphasizing the role of surface treatments in improving barrier function. Together, these findings suggest that while some implant surface characteristics may have limited influence on initial cell metabolic activity after specimen removal and residual viability and permeability, roughened or chemically modified surfaces, particularly those with UV treatment, promote enhanced soft tissue viability and barrier integrity, highlighting the importance of combined surface topography and treatment strategies for optimizing peri‐implant soft tissue integration.

**Table 9:** Permeability test assessing epithelial barrier function and integrity of 3D oral mucosa models surrounding various abutment materials.

Biological test: permeability test				
Article	Biological system	Implant	Methods	Results
Chai et al. [17]	3D‐OMM was constructed using TR146 and HGF cultured onto the basement membrane side of the acellular scaffold	1. Polished Ti 2. Machined Ti 3. Sandblasted Ti 4. Anodized Ti	HTO radioactivity	By the end of the experiment (Pa5), HTO penetration was less than 20% across all experimental models, demonstrating similar permeability and no significant differences
Razali et al. [1]	3D‐OMM was constructed using HGK and HGF cultured on an acellular dermal membrane	1. YSZ 2. ATZ 3. CPTi	HTO radioactivity	Untreated CPTi exhibited higher HTO penetration than other materials, while treated groups showed reduced penetration. Significant differences were found between N‐Tx and UV‐Tx treatments (*p* = 0.002). Among materials, YSZ differed significantly from CPTi (*p* < 0.001) and ATZ (*p* = 0.017), whereas no significant difference was observed between CPTi and ATZ (*p* = 0.061)

*Note:* HTO, tritiated water; N‐Tx, nontreated group; Pa5, final time point of the experiment; TR146, human buccal squamous cell carcinoma cell line; UV‐Tx, ultraviolet‐treated group.

Abbreviations: 3D‐OMM, three‐dimensional oral mucosa model; ATZ, alumina‐toughened zirconia; CPTi, commercially pure titanium; HGFs, human gingival fibroblasts; HGKs, human gingival keratinocytes; Ti, titanium; YSZ, yttria‐stabilized zirconia.

### 3.5. Molecular Biology and Microbiology

While qualitative analysis offers important insights into host–microbe interactions at the peri‐implant interface, incorporating quantitative measures such as cytokine profiling and gene expression analysis provides a deeper, more objective understanding of the underlying biological responses. This integrated approach strengthens the clinical relevance of in vitro findings and supports the development of more effective strategies for preventing and managing peri‐implant diseases.

The host–microbe interaction is critical in maintaining oral health and influencing the development of peri‐implant diseases. The peri‐implant mucosa and commensal biofilms are essential for preserving host–microbe homeostasis, yet their specific interactions remain poorly understood. Additionally, the effects of commensal and pathogenic oral bacteria on peri‐implant mucosa are not fully elucidated [25, 26]. Table 10 summarizes the different molecular approaches used to evaluate soft tissue response to different abutment materials. Ingendoh‐Tsakmakidis et al. [9] explored the responses of peri‐implant mucosa to biofilms formed by *Streptococcus oralis* (*S. oralis*) and *Aggregatibacter actinomycetemcomitans* (*A. actinomycetemcomitans*) using an in vitro 3D organotypic peri‐implant mucosa model. Findings revealed that exposure to *S. oralis* biofilm reduced inflammatory cytokine levels (IL‐6, IL‐8, IL‐1β, and CCL2), except for a significant increase in tumor necrosis factor alpha (TNF‐α), a key inflammatory mediator [9, 13]. Cytokines controlled by commensal bacteria limit biofilm development, consequently maintaining gingival health [27]. Conversely, exposure to *A. actinomycetemcomitans* biofilm further suppressed chemotactic and proinflammatory chemokines (CXCL1, CXCL8, and CCL2), facilitating biofilm expansion. Gene expression analysis via microarray indicated that *S. oralis* exposure was associated with reduced adaptive responses, promoting mucosal homeostasis. In contrast, *A. actinomycetemcomitans* exposure triggered gene expression related to DNA repair and cell division, suggesting a stress response that could enhance colonization and tissue invasion [9]. Despite that, these data aligned with other in vivo studies, yet responses on transcription and cytokine levels were uncovered. This explains why monospecies commensal and pathogenic biofilms do not cause significant inflammatory reactions. Instead, Mikolai et al. [10] investigated the impact of multispecies biofilm (*S. oralis*, *Actinomyces naeslundii*, *Veillonella dispar*, and *Porphyromonas gingivalis*) on 3D organotypic peri‐implant mucosa at different timepoints of exposure. After 24 h, biofilms induced a mild inflammatory response with reduced biofilm volume, potentially due to β‐defensins‐1/‐2 and CCL20 secretion. However, after 48 h of exposure, mucosal damage was observed alongside altered bacterial distribution and an intensified immune response marked by upregulated inflammatory genes and cytokine secretion (e.g., IL‐1β and TNF‐α) [10]. These findings indicate that prolonged biofilm exposure disrupts host–microbe homeostasis. Similarly, Souza et al. [21] investigated the mutualistic interactions between *Candida albicans* and Mitis group *Streptococcus* species (*S. mitis*, *S. oralis*, *S. sanguinis*, and *S. gordonii*) within polymicrobial biofilms on Ti surfaces and their effects on adjacent mucosal tissues using a 3D in vitro mucosal infection model. The study demonstrated that mixed‐species biofilms significantly enhanced tissue damage compared to monospecies *Candida* biofilms, which was linked to increased expression of fungal hypha–associated virulence genes. Despite this, the biofilms did not induce a heightened inflammatory cytokine response, suggesting a complex immunomodulatory interaction. In addition, interactions between the biofilms and mucosal tissues triggered the release of growth‐suppressing mediators targeting *C. albicans*, indicating a potential homeostatic defense mechanism against fungal overgrowth [21]. These findings provide novel insights into cross‐kingdom interactions, highlighting their role in the pathogenesis of peri‐implant mucosal infections and the interplay between biofilm virulence and host defense mechanisms at the biomaterial‐tissue interface. Moreover, understanding these host–microbe interactions at the peri‐implant site provides valuable insights for developing improved preventive and therapeutic strategies for peri‐implant diseases. A recent study by Llopis‐Grimalt et al. [24] investigated the potential of NN Ti surfaces to enhance soft tissue integration around dental implants to prevent peri‐implantitis by forming a long‐lasting transmucosal seal. The researchers evaluated the implant‐tissue interface response using a 3D gingival tissue equivalent (GTE) exposed to both NN Ti and machined Ti surfaces, simulating peri‐implantitis conditions with *P. gingivalis* lipopolysaccharide (LPS) stimulation. Interestingly, by employing enzyme‐linked immunosorbent assay (ELISA) and gene expression techniques, their results indicated no significant impact of NN Ti on the induction of metalloproteinase‐1 (MMP1) and its inhibitor (TIMP1), as seen by machined Ti implants. These proteins regulate collagen degradation and extracellular matrix turnover, and under inflammatory conditions, the MMP/TIMP (tissue inhibitor of metalloproteinase) ratio is upregulated, enhancing collagen degradation [28–31]. Although nonstatistically significant differences were found in gene expression analysis, increased expression levels of cell‐matrix turnover‐related genes (*COL1A1*, *COL3A1*, decorin (*DCN*), and *ACTA2*) and decreased expression levels of the proinflammatory cytokine IL‐6 for the NN Ti were observed. Such induction of collagen formation mimics the natural situation of collagen formation in the tooth in the gingival tissue [24]. Hence, highlighting the tissue biocompatibility of NN implants, allowing a better soft tissue sealing around dental implants and, in turn, preventing peri‐implantitis (Table 10). On the other hand, Jalaleddine et al. [14] utilized a sophisticated 3D oral mucosal model incorporating fibroblasts, keratinocytes, and THP‐1 monocytes to systematically compare inflammatory responses across implant materials. ELISA quantification of culture supernatants demonstrated that machined zirconia (ZrO_2_‐M) and PEEK‐M surfaces triggered markedly elevated IL‐1β secretion (*p*  < 0.0001) compared to sandblasted/large‐grit/acid‐etched TiZr‐SLA, machined TiZr‐M, and polished ZrO_2_‐P surfaces. Notably, IL‐6 and IL‐8 levels remained equivalent across all materials, establishing IL‐1β as the primary discriminator of material‐specific proinflammatory potential. These findings indicate that surface topography critically modulates acute inflammatory signaling in peri‐implant tissues (Table 10).

**Table 10:** Molecular biology analysis of gene and protein expression related to inflammation, tissue response, and soft tissue integration around different abutment materials.

Biological test: molecular biology				
Article	Biological system	Implant	Methods	Results
Ingendoh‐Tsakmakidis et al. [9]	3D‐OMM was constructed using HGFs on a collagen scaffold with OKF6/TERT‐2 cocultured with and without *S. oris* or *A. actinomycetemcomitans* biofilms	HGF‐colonized Ti disk	Microarray gene expression	24 h *S. oralis* exposure on peri‐implant 3D mucosa model: 83 genes differentially expressed: • 36 upregulated genes associated with HSP70, MAPK, and chemokine pathways (CCL20, CCL8, and PIK3R5). • 47 downregulated genes associated with HLA‐DRA of MHC class II 24 h *A. actinomycetemcomitans* exposure on peri‐implant 3D mucosa model: 101 genes were differentially expressed. • 32 upregulated genes associated with cell division (FIGN, HMGA2, CDC25A, and ERCC6L) and DNA repair/damage (CLSPN, POLQ, and FANCA) • 69 downregulated genes related to PI3K‐AKT pathway and other signaling transduction pathways (MDM2, IL2RG, TLR4, and F2R)
			Luminex‐based multiplex assay measuring proinflammatory cytokines and chemokines	24 h *S. oralis* exposure on peri‐implant 3D mucosa model: • Significant decrease in inflammatory response (IL‐6, IL‐8, IL‐1, and CCL‐2) while a significant increase in TNF‐α 24‐h *A. actinomycetemcomitans* exposure on peri‐implant 3D mucosa model: • Significant decrease of chemotactic and proinflammatory chemokines (CXCL1, CXCL8, and CCL2)
Mikolai et al. [10]	3D‐OMM was constructed using HGFs and OKF6/TERT‐2 on a collagen scaffold cocultured with and without multispecies biofilms: *S. oralis*, *A. naeslundii*, *V. dispar*, and *P. gingivalis* biofilm	HGF‐colonized Ti disk	Microarray gene expression	Peri‐implant embedded 3D mucosa model exposure to multispecies biofilm: 24 h exposure: • 5 upregulated genes involved in cytokine–cytokine receptor interaction pathways 48 h exposure: • 122 differentially expressed genes: • 14 upregulated genes (TNF‐α and cytokine interaction pathways) • 108 downregulated genes (cell adhesion pathways)
			Luminex‐based multiplex and ELISA assays measuring hBD and proinflammatory cytokines and chemokines	Peri‐implant embedded 3D mucosa model exposure to multispecies biofilm: • 24 h exposure: significant increase in IL‐6 and CCL20 • 48 h exposure: significant increase in IL‐1β, TNF‐α, and CCL20 • Decreased at both time points: CXCL2 • No significant change: CXCL1, CXCL8, CCL2, hBD‐1, and hBD‐2
Souza et al. [21]	3D‐OMM was constructed of SCC15 collagen scaffold and 3T3 cocultured with monospecies (*C. albicans*) or mixed‐species biofilms (*C. albicans* with different *Streptococcus* species)	Machined Ti discs	qRT‐PCR for hypha‐associated gene expression quantification	6 h coculture: • All *Streptococcus* species increased *EFG1* expression in *C. albicans* • *S. oralis* and *S. gordonii* upregulated *HWP1* and *als3* in mixed biofilms 24 h coculture: No significant changes in hypha‐associated genes (*als3*, *HWP1*, and *EFG1*)
			Luminex‐based multiplex assay measuring proinflammatory cytokines	Nonsignificant change in cytokines (IL‐1β, Il‐6, IL‐8, and TNF‐α) between 3D models juxtaposed to mixed‐species biofilms and 3D models cocultured with only *C. albicans*
Llopis‐Grimalt et al. [24]	3D‐OMM was constructed using GTE of iHGF and iHGK	NN Ti or machined Ti	RT‐PCR measuring genes of ECM components, wound healing, and proinflammatory cytokines	The NN Ti compared to machined Ti revealed: • Increased expression of: COL1A1, COL3A1, DCN, and ACTA2 • Decreased expression of: IL‐6
			ELISA measuring MMP1 and TIMP1	Nonsignificant effect on MMP1 production or its inhibitor TIMP1
Jalaleddine et al. [14]	3D‐OMM was composed of stratified oral keratinocytes (OKF6/TERT‐2) cultured atop a collagen‐based connective tissue populated with HGFs and THP‐1 monocytes	1. TiZr‐SLA 2. TiZr‐M 3. ZrO_2_‐M 4. PEEK‐M	ELISA measuring IL‐1β, IL‐6, and TNF‐α	ELISA quantification of supernatants demonstrated markedly elevated IL‐1β secretion from ZrO_2_‐M and PEEK‐M groups compared to TiZr‐SLA, TiZr‐M, and ZrO_2_‐P (*p* < 0.0001). IL‐6 and IL‐8 levels remained comparable across surfaces, identifying IL‐1β as the only primary proinflammatory marker

*Note:* 3T3, mouse‐derived fibroblasts; ACTA2, alpha‐smooth muscle actin; ALS3, agglutinin‐like protein 3; CXCL, C‐X‐C motif chemokine ligands; CCL2, C–C motif chemokine ligand 2; CDC25A, M‐phase inducer phosphatase 1; ERCC6L, DNA excision repair protein ERCC‐6‐like; F2R, coagulation factor II thrombin receptor; FANCA, Fanconi anemia group A protein; HLA‐DRA, HLA class II histocompatibility antigen DR alpha chain; OKF6/TERT‐2, immortalized human oral keratinocytes; PI3K‐AKT, phosphatidylinositol 3‐kinase/protein kinase B pathway; POLQ, DNA polymerase theta; SCC15, immortalized human oral keratinocytes.

Abbreviations: 3D‐OMM, three‐dimensional oral mucosa model; *A. actinomycetemcomitans*, *Aggregatibacter actinomycetemcomitans*; *A. naeslundii*, *Actinomyces naeslundii*; *C. albicans*, *Candida albicans*; CLSPN, claspin; COL, collagen; DCN, decorin; EFG1, enhanced filamentous growth protein 1; ELISA, enzyme‐linked immunosorbent assay; FIGN, fidgetin, GTE, gingival tissue equivalent; HMGA2, high mobility group protein A2; hBD, human beta‐defensin; HGFs, human gingival fibroblasts; h, hour; HSP70, heat shock protein 70, HWP1, hyphal wall protein 1; iHGF, immortalized human gingival fibroblasts (hTERT); iHGK, immortalized human gingival keratinocytes (Gie‐No3B11); ILs, interleukins; MAPK, mitogen‐activated protein kinase; MDM2, murine double minute 2; IL2RG, interleukin 2 receptor gamma; NN Ti, nanostructured titanium; *P. gingivalis*, *Porphyromonas gingivalis*; qRT‐PCR, quantitative reverse transcription PCR; *S. oralis*, *Streptococcus oralis*; Ti, titanium; TIMP, tissue inhibitor of metalloproteinases; TLR4, toll‐like receptor 4; TNF‐α, tumor necrosis factor alpha; *V. dispar*, *Veillonella dispar*.

Microbiological studies using 3D‐OMMs have also investigated the interactions between peri‐implant biofilms and soft tissues on Ti surfaces (Table 11). Mikolai et al. [10] demonstrated that exposure of multispecies biofilms to peri‐implant mucosa reduced overall biofilm volume after 24 and 48 h, with shifts in bacterial composition favoring increased proportions of *S. oralis* and *V. dispar* and decreased *A. naeslundii* after 48 h. Souza et al. [21] reported that *S. oralis* formed more extensive biofilms on machined Ti than other bacteria, and mixed biofilms of *S. oralis* with *C. albicans* exhibited larger biovolumes compared to combinations with other streptococci. Notably, *C. albicans* growth was inhibited by spent media from biofilm‐exposed mucosal tissues, particularly in single and mixed biofilms with *S. gordonii*, while the same media promoted *S. oralis* growth; media from biofilms without mucosal exposure had no effect (Table 11) [21]. These findings highlight the dynamic influence of peri‐implant mucosa on biofilm composition and behavior, emphasizing the complex interplay between soft tissues and microbial communities in modulating biofilm development and pathogenic potential around implants.

**Table 11:** Microbiological analysis assessing bacterial adhesion, biofilm formation, and microbial interactions on various abutment materials.

Biological test: microbiology				
Article	Biological system	Implant	Methods	Results
Mikolai et al. [10]	3D oral mucosa model was constructed using HGFs and OKF6/TERT‐2 on a collagen scaffold cocultured with and without multispecies biofilms: *S. oralis*, *A. naeslundii*, *V. dispar*, and *P. gingivalis* biofilm	HGF‐colonized Ti disk	LIVE/DEAD BacLight Bacterial Viability	Reduction of biofilm volume after peri‐implant mucosa exposure after 24 and 48 h of exposure
			FISH staining: fluorescence in situ hybridization	Bacterial distribution: • After 24 h: exposure to the mucosa had no impact on bacterial distribution • After 48 h: a significantly higher volume proportion of *S. oralis* and *V. dispar* was observed, and a significantly lower volume proportion of *A. naeslundii* than in the 48 h control biofilms
Souza et al. [21]	3D‐OMM was constructed of SCC15 collagen scaffold and 3T3 cocultured with monospecies (*C. albicans*) or mixed‐species biofilms (*C. albicans* with different *Streptococcus* species)	Machined Ti discs	qPCR via 16S rRNA for biomass quantification after 72 h coculturing	• *S. oralis* formed more extensive biofilms on Ti discs than other bacteria when cocultured individually with embedded mucosa • Mixed biofilms of *S. oralis* with *C. albicans* had a larger biovolume than mixed biofilms with other streptococcal species • *C. albicans* biovolumes were negatively affected by all streptococci except for *S. oralis*
			Effect of spent media from biofilms on *C. albicans* growth on Ti using biofilms	• Spent culture media concentrations (0% to 20%) from biofilm‐exposed tissues increasingly inhibited *C. albicans* growth on Ti surfaces, both in single and mixed biofilms with *S. gordonii* • Spent media from these tissues promoted the growth of *S. oralis* but did not affect other *Streptococcus* species • Biofilm spent media collected without a mucosal construct did not impact *C. albicans* growth in single or mixed biofilms on Ti surfaces

Abbreviations: 3D‐OMM, three‐dimensional oral mucosa model; *C. albicans*, *Candida albicans*; HGFs, human gingival fibroblasts; h, hour; rRNA, ribosomal ribonucleic acid; RT‐PCR, real‐time reverse transcription polymerase chain reaction; *S. gordonii*, *Streptococcus gordonii*; *S. oralis*, *Streptococcus oralis*; Ti, titanium.

## 4. Discussion

The systematic review evaluated biological tests and methodologies for assessing implant abutment–soft tissue attachment in 3D tissue–engineered oral mucosa models. The QUIN assessment suggests that the included studies were generally well reported in several domains, particularly aims, comparison groups, methodology, and outcome presentation. However, reporting of randomization and blinding was inconsistent, which should be considered when interpreting the findings. Overall, most included studies were judged to have a low‐to‐moderate risk of bias, primarily due to incomplete reporting of randomization and blinding.

The included studies provide important insights into biological interactions at the peri‐implant soft tissue interface across implant materials and surface modifications. Immunohistochemical analyses show that engineered mucosa recapitulates key epithelial differentiation markers and basement membrane components, while implant surface properties influence keratin expression and collagen fiber organization [4, 18, 19, 22, 24]. SEM and TEM show that epithelial cells adhere robustly to Ti and ceramic surfaces regardless of topography, although roughness and treatments such as anodization and UV irradiation modulate cell morphology, viability postpull‐out test, and ultrastructural integration via hemidesmosome‐like contacts [4, 8, 17]. AFM confirms that implant materials generally exhibit similar smoothness at the nanoscale, highlighting the primacy of surface chemistry and modifications in affecting cellular responses [1].

Functional evaluations through postpull‐out metabolic activity and permeability assays yielded mixed findings. Some studies reported negligible differences in residual viability across surfaces, whereas others found improved barrier function on roughened or surface‐treated implants, particularly with hydrophilic modifications such as UV treatment [1, 17, 20, 24]. Our recent investigation [14] confirmed these findings, showing that UV‐photofunctionalized Ti significantly enhanced epithelial down‐growth and peri‐implant seal formation compared with machined Ti controls (42% improvement, *p*  < 0.01). This validation strengthens the trend toward hydrophilic and UV‐modified surfaces across the included studies and supports biomaterial optimization for improved soft tissue integration [14]. Microbiological investigations further underscore the critical role of peri‐implant mucosa in shaping biofilm composition and behavior, revealing dynamics in bacterial species dominance and fungal growth modulation contingent upon mucosal interactions and the surrounding microenvironment [10, 21].

Despite significant progress, notable gaps remain. Engineered mucosal models often lack full replication of native tissue complexity, including vascularization, which may limit fidelity in simulating in vivo conditions. Additionally, while surface topography and chemistry clearly influence soft tissue integration, the precise molecular mechanisms governing these effects warrant deeper exploration. The interplay between immune components, microbial biofilms, and peri‐implant tissues is only beginning to be elucidated, suggesting a need for more integrative models incorporating host immune responses.

In summary, the available in vitro evidence suggests that implant surface engineering may influence epithelial attachment, extracellular matrix organization, and barrier function, but the studies are heterogeneous and do not support definitive comparative conclusions. While these 3D‐OMMs provide valuable mechanistic insight, they do not fully recapitulate clinical implant–abutment interface dynamics, including the influence of connection design on soft tissue sealing [32]. Future research should focus on refining 3D‐OMMs to mimic native architecture and immune environments better and on characterizing molecular pathways mediating host–material–microbe interactions to inform rational design of implants that minimize peri‐implant disease risk and improve clinical outcomes.

## 5. Conclusions

This systematic review highlights that 3D tissue–engineered oral mucosa models are valuable in vitro platforms for studying implant abutment–soft tissue interactions. The available evidence suggests that some surface modifications may be associated with more favorable epithelial and connective tissue responses, but the findings are heterogeneous and endpoint‐dependent. Overall, Ti‐based and modified surfaces frequently showed favorable trends in several assays, whereas comparisons with zirconia and PEEK varied across models and outcomes. Permeability and postpull‐out metabolic activity results were not uniform across studies, underscoring the need for caution in interpretation. Future work should prioritize standardized models and more complete biological systems to improve comparability and translational relevance.

## 6. Limitations and Future Directions

This systematic review synthesized findings from 14 heterogeneous in vitro studies, limiting direct comparison across models and preventing formal meta‐analysis. Differences in 3D culture systems, endpoint measures, implant surfaces, and surface modifications also limited cross‐study comparisons and precluded definitive ranking of material or topography effects. In addition, current in vitro models do not fully reproduce the clinical oral environment, including vascularization, biomechanical loading, and the complexity of the oral microbiome and the influence of implant abutment connection design on soft tissue sealing. Future studies should focus on standardized models, immune and vascular incorporation, and better characterization of host–material–microbe interactions to strengthen the evidence base for future translational and clinical work.

## Author Contributions

Conceptualization: Keyvan Moharamzadeh, Moosa Abuzayeda, and Rawan Aboud. Methodology: Keyvan Moharamzadeh, Moosa Abuzayeda, Rawan Aboud, and Nour Jalaleddine. Software: Rawan Aboud and Nour Jalaleddine. Validation: Nour Jalaleddine and Keyvan Moharamzadeh. Formal analysis: Rawan Aboud. Investigation: Moosa Abuzayeda, Rawan Aboud, Nour Jalaleddine, and Dalia Alsadig Mohamed. Resources: Keyvan Moharamzadeh and Moosa Abuzayeda. Data curation: Nour Jalaleddine and Dalia Alsadig Mohamed. Writing – original draft preparation: Rawan Aboud and Nour Jalaleddine. Writing – review and editing: Nour Jalaleddine, Rawan Aboud, Dalia Alsadig Mohamed, Keyvan Moharamzadeh, and Moosa Abuzayeda. Visualization: Nour Jalaleddine and Rawan Aboud. Supervision: Nour Jalaleddine. Project administration: Keyvan Moharamzadeh, Moosa Abuzayeda, and Nour Jalaleddine. Funding acquisition: Keyvan Moharamzadeh and Moosa Abuzayeda.

## Funding

This research was supported by an Internal Research Grant from Hamdan Bin Mohammed College of Dental Medicine (HBMCDM), Mohammed Bin Rashid University of Medicine and Health Sciences (MBRU) (Grant MBRU‐HBMCDM‐RG2025‐01).

## Disclosure

All authors have read and agreed to the published version of the manuscript.

## Ethics Statement

The authors have nothing to report.

## Consent

The authors have nothing to report.

## Conflicts of Interest

The authors declare no conflicts of interest.

## Supporting Information

Additional supporting information can be found online in the Supporting Information section.

## Supporting information


**Supporting Information 1** Table S1: Detailed search strategy, including the keywords and Boolean operators used across the electronic databases to identify all potentially eligible studies.

**Supporting Information 2** Table S2: List of full‐text articles excluded after eligibility assessment, with the specific reasons for exclusion reported for each study.

## Data Availability

The original contributions presented in the study are included in the article; further inquiries can be directed to the corresponding author.
