# Supplementary material for: Evaluation of Implant Abutment–Soft Tissue Attachment Using 3D Tissue‐Engineered Oral Mucosa: A Systematic Review
Source: Int J Dent. 2026 Jun 27;2026:5005401. doi: 10.1155/ijod/5005401 (PMC13309904; doi:10.1155/ijod/5005401)
Supplement: Supplementary file 1 — Supporting Information 1 Table S1: Detailed search strategy, including the keywords and Boolean operators used across the electronic databases to identify all potentially eligible studies. [file IJOD-2026-5005401-s002.docx]

| **Database/source** | **Exact search string** | **Date searched** | **Limits/filters** |
| --- | --- | --- | --- |
| PubMed/MEDLINE | (“3D oral mucosa” OR organotypic OR “tissue-engineered oral mucosa” OR “oral mucosa equivalent” OR “oral mucosal model”) AND (“dental implant” OR implant* OR abutment* OR “implant abutment”) AND (“soft tissue” OR “soft tissue interface” OR “peri-implant mucosa” OR “mucosal seal”) | 31 Mar 2026 | English; 2010–2026 |
| Scopus | TITLE-ABS-KEY ( “3D oral mucosa” OR organotypic OR “tissue-engineered oral mucosa” OR “oral mucosa equivalent” OR “oral mucosal model” ) AND TITLE-ABS-KEY ( “dental implant” OR implant* OR abutment* OR “implant abutment” ) AND TITLE-ABS-KEY ( “soft tissue” OR “soft tissue interface” OR “peri-implant mucosa” OR “mucosal seal” ) | 31 Mar 2026 | English; 2010–2026 |
| Embase | ('3d oral mucosa':ti,ab OR organotypic:ti, ab OR 'tissue engineered oral mucosa':ti,ab OR 'oral mucosa equivalent':ti,ab OR 'oral mucosal model':ti,ab) AND ('dental implant':ti,ab OR implant*:ti,ab OR abutment*:ti,ab OR 'implant abutment':ti,ab) AND ('soft tissue':ti,ab OR 'soft tissue interface':ti,ab OR 'peri-implant mucosa':ti,ab OR 'mucosal seal':ti,ab) | 31 Mar 2026 | English; 2010–2026 |
| Google Scholar | "3D oral mucosa" implant abutment soft tissue | 31 Mar 2026 | First relevant pages screened |
| Google | "3D oral mucosa" implant abutment soft tissue | 31 Mar 2026 | Grey literature / supplementary search |
| Author publication histories | Relevant authors in this field | 31 Mar 2026 | Manual screening |
| Reference lists | Reference lists of included studies and reviews | 31 Mar 2026 | Hand-search |

**Supplementary Table S1.** Detailed search strategy, including the keywords and Boolean operators used across the electronic databases to identify all potentially eligible studies.
