# Supplementary material for: Evaluation of Implant Abutment–Soft Tissue Attachment Using 3D Tissue‐Engineered Oral Mucosa: A Systematic Review
Source: Int J Dent. 2026 Jun 27;2026:5005401. doi: 10.1155/ijod/5005401 (PMC13309904; doi:10.1155/ijod/5005401)
Supplement: Supplementary file 2 — Supporting Information 2 Table S2: List of full‐text articles excluded after eligibility assessment, with the specific reasons for exclusion reported for each study. [file IJOD-2026-5005401-s001.docx]

|  | Inclusion Criteria | Exclusion Criteria |
| --- | --- | --- |
| Cells at the implant interface | 3D cultured cells or cell lines from donor tissue of human oral mucosa origin; Human oral mucosa cells (keratinocytes/fibroblasts) form functional tissue-dental implant contact. | Animal cells ONLY (no human cells at interface). |
| Study Type | Implant and abutment insertion in 3D tissue-engineered model- in vitro 3D organotypic models. | In vivo and clinical studies. |
| Feeder Layers | Xenogeneic feeders OK (3T3-J2 mitomycin-C inactivated). | - |
| Language | Publications written in English. | Publications not written in English |

**Supplementary Table S2.** List of full-text articles excluded after eligibility assessment, with the specific reasons for exclusion reported for each study.
